# Ethylene Biosynthesis Inhibition Combined with Cyanide Degradation Confer Resistance to Quinclorac in *Echinochloa crus-galli* var. *mitis*

**DOI:** 10.3390/ijms21051573

**Published:** 2020-02-25

**Authors:** Muhammad Zia Ul Haq, Zheng Zhang, Jiajia Wei, Sheng Qiang

**Affiliations:** 1 Weed Research Laboratory, College of Life Sciences, Nanjing Agricultural University, Nanjing 210095, China; ziaagr@yahoo.com (M.Z.U.H.); jaycheung2009@163.com (Z.Z.); yinweier@yeah.net (J.W.); 2 Department of Agronomy, University of Agriculture, Faisalabad 38000, Pakistan

**Keywords:** *Echinochloa crus-galli* var. *mitis*, ethylene biosynthesis inhibition, quinclorac resistance, ACC synthase and ACC oxidase, ACS and ACO genes, *β*-cyanoalanine synthase

## Abstract

*Echinochloa crus-galli* var. *mitis* has rarely been reported for herbicide resistance, and no case of quinclorac resistance has been reported so far. Synthetic auxin-type herbicide quinclorac is used extensively to control rice weeds worldwide. A long history of using quinclorac in Chinese rice fields escalated the resistance in *E. crus-galli* var. *mitis* against this herbicide. Bioassays in Petri plates and pots exhibited four biotypes that evolved into resistance to quinclorac ranking as JS01-R > AH01-R > JS02-R > JX01-R from three provinces of China. Ethylene production in these biotypes was negatively correlated with resistance level and positively correlated with growth inhibition. Determination of the related ethylene response pathway exhibited resistance in biotypes that recorded a decline in 1-aminocyclopropane-1-carboxylic acid (ACC) content, ACC synthase oxidase activities, and less inducible *ACS* and *ACO* genes expressions than the susceptible biotype, suggesting that there was a positive correlation between quinclorac resistance and ethylene biosynthesis inhibition. Cyanides produced during the ethylene biosynthesis pathway mainly degraded by the activity of *β*-cyanoalanine synthase (*β*-CAS). Resistant biotypes exhibited higher *β*-CAS activity than the susceptible ones. Nucleotide changes were found in the *EcCAS* gene of resistant biotypes as compared to sensitive ones that caused three amino acid substitutions (Asn-105-Lys, Gln-195-Glu, and Gly-298-Val), resulting in alteration of enzyme structure, increased binding residues in the active site with its cofactor, and decreased binding free energy; hence, its activity was higher in resistant biotypes. Moreover, these mutations increased the structural stability of the enzyme. In view of the positive correlation between ethylene biosynthesis inhibition and cyanide degradation with resistance level, it is concluded that the alteration in ethylene response pathway or at least variation in ACC synthase and ACC oxidase enzyme activities—due to less relative expression of *ACS* and *ACO* genes and enhanced *β*-CAS activity, as well as mutation and increased relative expression of *EcCAS* gene—can be considered as a probable mechanism of quinclorac resistance in *E. crus-galli* var. *mitis*.

## 1. Introduction

Contributing more than USD 34 billion toward herbicidal control cost [1], weeds are considered to be the most troublesome entity in agricultural systems [2]. Weed control by using herbicides dates back to 1945 with the commercial availability of 2,4-dichlorophenoxyacetic acid (2,4-D (synthetic auxin)) [3]. Herbicides greatly contributed to abundant food production worldwide. Nevertheless, the evolution of a resistant population is a major threat to the sustained efficacy of these herbicides. Robust selection pressure, which is imposed by herbicides on vast and genetically assorted pest populations, firstly results in high mortality; on the other hand, selection and enrichment of the rare resistant genes present in the population result in the evolution of resistance [4,5]. Worldwide, 256 species containing 500 resistant biotypes are present, which consists of 149 dicotyledon and 107 monocotyledon biotypes [6].

Selection for multiple herbicide-resistant weed populations stems from the long history of herbicide use [6], eventually leaving fewer options for effectual alternative herbicides. The mechanisms attributing to herbicide resistance in weeds are target-site resistance (TSR) and non-target-site resistance (NTSR). Herbicide resistance can occur by the alteration of the gene that translates the herbicide target protein. Even a single nucleotide substitution in genes is enough to cause herbicide resistance [7]. Additionally, copy number variation [8], codon triplet deletion [9], target-site protein over-production, and change in the structure of binding site by amino acid substitution [10] have been reported so far, and these are characterized as TSR because this is caused by herbicide target gene mutation. Any mechanism causing a change in herbicide absorption, translocation, metabolism, and degradation are categorized as NTSR [11]. Genes involved in NTSR mechanism often comprise a superfamily, hence prompting difficulty in gene identification [12]. Consequently, a fewer number of genes have been reported for NTSR [13,14,15]. In outcrossing species, gene flow of resistance alleles and sequential herbicide selection can bring several mechanism accumulations rendering multiple herbicide resistance [16].

In Chinese rice fields, barnyard grass (*Echinochloa crus-galli*) is considered as the most problematic weed [17,18,19], and it is ranked as the sixth most troublesome herbicide-resistant weed globally [20]. *Echinochloa crus-galli* is much devastating that it can reduce the rice biomass and yield by 75% and 50%, respectively, even at a 10:1 ratio of rice and *E. crus-galli* plants [21]. To control *Echinochloa* spp. in rice fields, herbicides are being applied for more than 30 years. Nonetheless, herbicide resistance is rapidly developing due to the persistent use of herbicides [7]. Eight herbicide-resistant species have been reported from genus *Echinochloa* in different countries around the globe [6].

Quinclorac with a quinolone carboxylic acid backbone is a synthetic auxin herbicide and is considered as an effective tool to control *Echinochloa* weed species [22,23,24]. Though, extensive use of this herbicide gave rise to quinclorac-resistant *Echinochloa* species such as *E. colona* [25,26], *E. crus-galli* [26,27,28,29], *E. crus-galli* var. *zelayensis* [18], *E. crus-pavonis* [6], *E. phyllopogon* [30], *E. oryzicola*, and *E. hispidula* [31].

TIR1/AFB has been discovered as an auxin receptor, as well as the receptor for quinclorac [23,32]. Quinclorac application results in the induction of auxin-responsive genes, including 1-aminocyclopropane-1-carboxylic acid (ACC) synthase, inducing the de novo synthesis of ACC synthase. In susceptible species, quinclorac application results in an increase of ACC, and then this produced ACC is oxidized into ethylene and hydrogen cyanide (HCN) by ACC oxidase. Several metabolic enzymes can be affected by a higher level of HCN, ultimately causing the death of susceptible plants [23]. One of the essential enzymes in the respiratory chain, cytochrome c oxidase, is inhibited by HCN [33]. A multigene family encodes the ACS (1-aminocyclopropane-1-carboxylic acid synthase) and ACO (1-aminocyclopropane-1-carboxylic acid oxidase) enzymes in the ethylene biosynthesis pathway [34]. Differential expressions of ACO and ACS genes were reported in quinclorac sensitive and resistant *E. crus-galli* var. *zalayensis* [19]. HCN produced during the ethylene biosynthesis was degraded by the activity of *β*-cyanoalanine synthase (*β*-CAS) [35]. Numerous studies mentioned that quinclorac resistance could be instigated by elevated levels of *β*-CAS activity [29,36,37]. Contrarily, quinclorac resistance in *E. phyllopogan* might solely depend on ethylene biosynthesis inhibition rather than cyanide degradation [38]. However, it is a fact that mutations can increase enzyme activity [39]. *β*-CAS mainly depends upon pyridoxal phosphate (PLP) for its activity [35]. Quinclorac-resistant *E. crus-galli* var. *zelayensis* was found to evolve single nucleotide mutation in *β*-CAS [37], but the effect of this mutation on enzyme structure stability and its binding with cofactor PLP needs further investigations.

Previous studies documented that there is no difference in quinclorac absorption/uptake, transport, and metabolism in resistant and susceptible plants [36,40,41]. Additionally, quinclorac did not cause cellulose synthesis inhibition [42]. The resistant populations of *E. crus-galli* var. *zelayensis* did not show a remarkable increase in ethylene level after quinclorac spray [18]. *Echinochloa crus-galli* var. *mitis* was found to evolve resistance against ALS-inhibiting herbicides such as imazethapyr + imazapic, penoxsulam, and bispyribac-sodium [43], as well as against pretilachlor [17]. Considering the unique mode of action of quinclorac, the TSR and NTSR mechanisms might be involved in its resistance. The low level of ACC synthase activity in resistant biotypes can be attributed to less sensitivity at auxin receptors level or/and altered auxin signal transduction pathway. Cyanide degradation by *β*-CAS can give further advantage to resistant biotypes to cope with quinclorac sensitivity.

*Echinochloa crus-galli* var. *mitis* is an important and widespread weed in China that has never been reported for quinclorac resistance. Four *E. crus-galli* var. *mitis* biotypes from different provinces of China were found to evolve resistance against quinclorac. To reveal the possible resistance mechanism, ethylene production and related enzymes such as ACC accumulation, ACS and ACO activities, *β*-CAS activity, its structure stability, and binding with cofactor PLP, as well as genes expression patterns, were comparatively evaluated between four resistant and one susceptible biotypes of *E. crus-galli* var. *mitis*.

## 2. Results

### 2.1. Dose–Response Analysis

**Figure 1:**
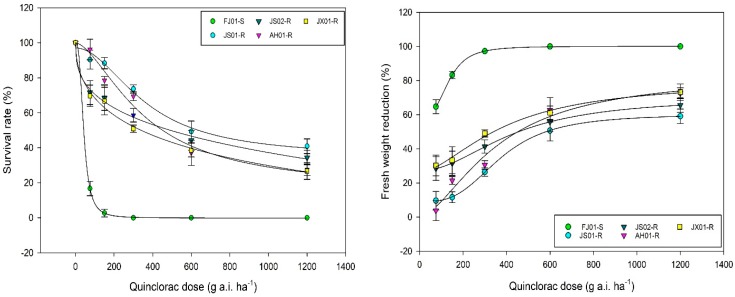
Dose–response curve of quinclorac-susceptible and resistant biotypes of *Echinochloa crus-galli* var. *mitis*. Survival rate (left) and fresh weight reduction (right; %) were measured three weeks after quinclorac application (0, 75, 150, 300, 600, and 1200 g a.i. ha^−1^). Dose–response curve was generated in Sigma Plot v.14.0 by employing logistic equation (four parameters).

**Figure 2:**
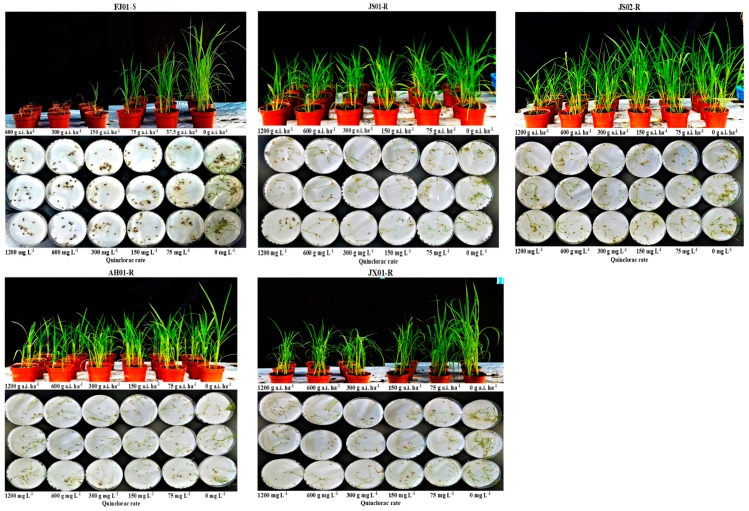
Quinclorac induced growth reduction (3 weeks after spraying in pot assay and 9 days after incubating the seeds with the quinclorac solution in Petri plate assay) in four resistant and one susceptible biotypes of *Echinochloa crus-galli* var. *mitis*.

In the seed and whole-plant bioassay, S biotype FJ01-S was unable to survive the increased rates of quinclorac, resulting in decreased EC_50_, GR_50,_ and RI (Table 1; Figure 1 and Figure 2). JX01-R biotype sustaining the quinclorac field rates proved resistant as compared to FJ01-S, as it showed a 12–14-fold increase in RI as compared to FJ01-S. However, AH01-R and JS02-R biotypes showed a remarkable increase in EC_50_, GR_50,_ and RI (Table 1); hence, these were proved more resistant than JX01-R. According to both assay results, JS01-R was dominated as a highly resistant biotype, as it exhibited a 27–35-fold increase in RI than FJ01-S biotype. The resistance level between all biotypes can be ranked as JS01-R > AH01-R > JS02-R > JX01-R.

### 2.2. Effect on Ethylene Biosynthesis

**Figure 3:**
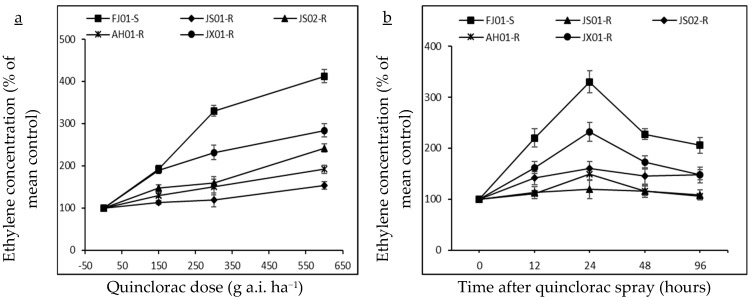
Ethylene concentration (% of mean untreated control) in quinclorac-resistant and susceptible biotypes of *Echinochloa crus-galli* var. *mitis* as influenced by different quinclorac dose rates (**a**; 0, 150, 300, and 600 g a.i. ha^−1^) at 24 h after treatment (HAT) and sampling times (**b**; 0, 12, 24, 48, and 96 HAT) at 300 g a.i. ha^−1^ quinclorac treatment. Vertical bars above mean represents the standard error of four replicates.

**Figure 4:**
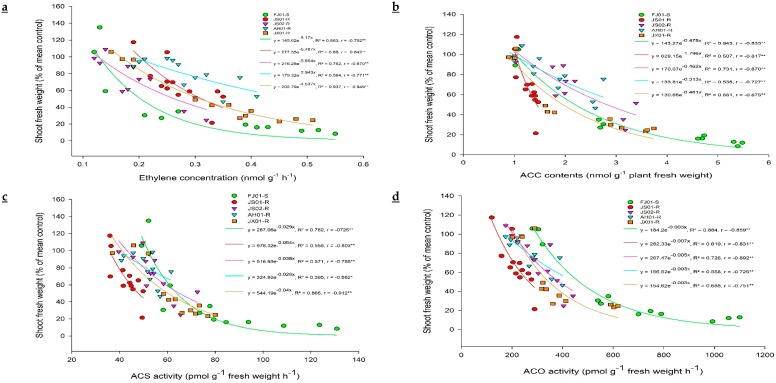
The correlation between (**a**) ethylene production, (**b**) 1-aminocyclopropane-1-carboxylic acid (ACC) contents, (**c**) 1-aminocyclopropane-1-carboxylic acid synthase (ACS) activity, (**d**) 1-aminocyclopropane-1-carboxylic acid oxidase (ACO) activity, and fresh weight (% of mean control) of *Echinochloa crus-galli* var. *mitis* plants after the application of 0, 150, 300, 600 g a.i. ha^−1^ quinclorac. Plant fresh weight was calculated 7 days after quinclorac application, and ethylene concentration, ACC content, ACS, and ACO activities were determined 24 h after quinclorac application. *R*^2^ = coefficient of determination, *r* = correlation coefficient, * and ** represent the correlation significance at *p* ≤ 0.05 and 0.01, respectively.

Quinclorac induces ethylene biosynthesis in susceptible plants. Hence, ethylene levels were measured in all biotypes to figure out its relationship with quinclorac resistance. Ethylene levels increased with the increasing rate of quinclorac in all biotypes, measured 24 h after spray (Figure 3a). However, this increment was not the same in all biotypes, as JS01-R exhibited a slight increase in ethylene level as compared to respective control (ethylene level without quinclorac spray) even at the highest quinclorac dose (600 g a.i. ha^−1^). FJ01-S, (quinclorac-susceptible biotype) showed a remarkable increase in ethylene levels on increased quinclorac rates. As this biotype exhibited 4.1 times increment in ethylene level at 600 g a.i ha^−1^ quinclorac treatment than the untreated control. On the same quinclorac rate, ethylene level increment in AH01-R, JS02-R, and JX01-R was 1.9, 2.4, and 2.8 times, respectively as compared to untreated control proving that ethylene production is negatively correlated with quinclorac resistance (Figure 4a). Ethylene production peaked after 24 h of quinclorac spray, and it started to decrease gradually later on in all biotypes at 300 g a.i. ha^−1^ quinclorac treatment (Figure 3b). However, the ethylene level measured in FJ01-S 96 h after spray on the same treatment level was 2 times higher than the untreated control. Moreover, quinclorac induced ethylene production caused toxicity in plants and resulted in fresh weight reduction. A positive correlation was found between growth inhibition and stimulation of ethylene production (Figure 4a). After quinclorac treatment, biotypes were ranked as FJ01-S > JX01-R > JS02-R > AH01-R > JS01-R regarding ethylene production, exhibiting a negative correlation between ethylene production and resistance levels (Figure 4a).

### 2.3. Effect on ACC (Precursor for Ethylene Biosynthesis)

**Figure 5:**
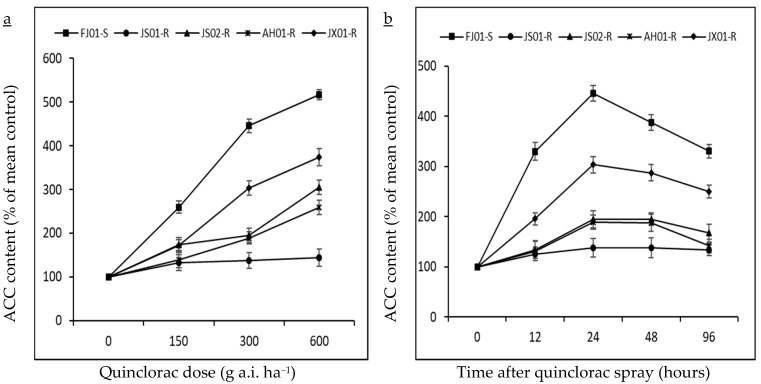
ACC content (% of mean untreated control) in quinclorac-resistant and susceptible biotypes of *Echinochloa crus-galli* var. *mitis* as influenced by different quinclorac dose rates (**a**; 0, 150, 300, and 600 g a.i. ha^−1^) at 24 HAT and sampling times (**b**; 0, 12, 24, 48, and 96 HAT) at 300 g a.i. ha^−1^ quinclorac treatment. Vertical bars above mean represents the standard error of four replicates. ACC = 1-Aminocyclopropane−1-carboxylic acid.

Ethylene production can be calculated directly by employing the endogenous ACC production rate, as a radical increase in ethylene production was recorded after exogenous ACC application in many plant tissues [44]. ACC accumulation (precursor of ethylene biosynthesis) was investigated in different biotypes of *E. crus-galli* var. *mitis* at 24 h after treating with varying rates of quinclorac (0, 150, 300, and 600 g a.i. ha^−1^; Figure 5a). ACC content increment was negligible in JS01-R, while other biotypes showed higher ACC content with increasing quinclorac rate. FJ01-S (quinclorac-susceptible biotype) recorded the highest ACC content (five times higher than untreated control) at 600 g a.i. ha^−1^ quinclorac rate (Figure 5a). While having a look at ACC content at 300 g a.i. ha^−1^ quinclorac rate, it is clear that its content increased till 24 h after spray but decreased afterward (Figure 5b). JS01-R showed a slight increase in ACC content regardless of the sampling time. Afterward, 24 h later, quinclorac application ACC contents were 4.4 and 3 times higher than untreated control in FJ01-S and JX01-R biotypes, respectively, while AH01-R and JS02-R biotypes exhibited 1.8 times higher ACC content than untreated control at the same sampling time (Figure 5b). ACC content in all biotypes followed the same trend as for ethylene levels and was negatively correlated with quinclorac resistance (Figure 4b).

### 2.4. ACS and ACO Enzymes Activity

In the ethylene biosynthesis pathway, ACS and ACO are two vital enzymes controlling the ethylene production rate. Hence, to find out the differential response of *E. crus-galli* var. *mitis* biotypes regarding quinclorac induced ACS and ACO enzyme activities, plants were treated with varying rates of quinclorac (0, 150, 300, and 600 g a.i. ha^−1^) till 24 h. A negative correlation was found among quinclorac resistance and ACS and ACO activities (Figure 4c,d).

#### 2.4.1. Effect on ACS Activity

ACS activity was higher in FJ01-S biotypes as compared to other biotypes without quinclorac treatment (Table 2). There was no significant difference in ACS activity in JS01-R biotype regardless of quinclorac rate. All other biotypes possessed a remarkable increment in ACS activity at increasing quinclorac rates (Table 2). FJ01-S biotype exhibited the highest (120% over mean untreated control) ACS activity at 600 g a.i. ha^−1^ quinclorac rate. While JX01-R, JS02-R, and AH01-R biotypes exhibited 68%, 47%, and 30% increase in ACS activity, respectively, at the same quinclorac rate (Table 2).

#### 2.4.2. Effect on ACO Activity

As ACS activity, ACO activity was higher in FJ01-S biotype before treating with quinclorac (Table 3). All the biotypes depicted differential increment in ACO activity at higher quinclorac rates. As JS01-R biotype exhibited the least increase (74% over untreated control) in ACO activity at 600 g a.i. ha^−1^ quinclorac rate. While JX01-R, JS02-R, and AH01-R biotypes showed 151, 81, and 72% increment than untreated control at the same quinclorac rate. FJ01-S (quinclorac-susceptible biotype) showed the highest ACO activity, as 77%, 147%, and 247% increase in ACO activity was recorded at 150, 300, and 600 g a.i. ha^−1^ quinclorac rate (Table 3).

### 2.5. Effect on β-CAS Activity

**Figure 6:**
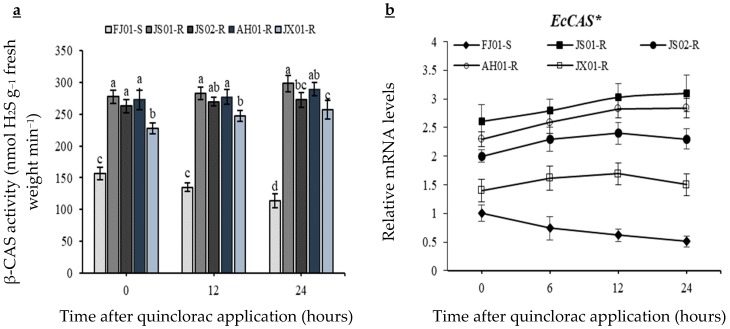
*β*-CAS activity (**a**), and *EcCAS* gene relative expression levels (**b**) measured at different time intervals after quinclorac application from five biotypes of *Echinochloa crus-galli* var. *mitis*. Data are mean of four biological replicates, and significance (*p* ≤ 0.05) at the same time level is described by (a–d) regarding the *β*-CAS activity (**a**). *X*-and *Y*-axis represents the time after quinclorac application and relative transcription level to susceptible biotype (0 h, prior to quinclorac application), respectively. Data presented are the mean of three biological and four technical replicates, and * represents upregulation more than 3-fold regarding *EcCAS* gene relative expression levels. Vertical bars above mean represent the standard error (**b**).

The significantly lower *β*-CAS activity was recorded in susceptible biotype as compared to resistant ones before quinclorac application (Figure 6a). Susceptible biotype (FJ01-S) exhibited a decreasing trend regarding *β*-CAS activity after quinclorac application, as a 25% decline in *β*-CAS was observed 24 h after treatment (HAT). Enzyme activity in resistant biotypes was significantly (*p* < 0.05) higher than susceptible one at all observed time periods (Figure 6a). JX01-R exhibited lower enzyme activity as compared to other resistant biotypes at all observed time periods, and at 24 HAT, JS02-R was at the same level than this biotype regarding the *β*-CAS activity. Slight *β*-CAS activity upsurge was observed in all resistant biotypes.

### 2.6. EcCAS Gene Sequence Description, Comparison, and Mutation Identification

After partial amplification of the *EcCAS* gene, the full sequence was obtained by amplifying 5′ and 3′ ends. The *EcCAS* gene ORFs (open reading frame) in five biotypes were sequenced, which consisted of 1113 bp, and it encoded 370 amino acids polypeptide and submitted to NCBI website (MN815009 and MN815010). The high similarity of the *EcCAS* gene from *E. crus-galli* var. *mitis* with the same gene in *E. crus-galli* var. *zelayensis* (KY922855.1; 99%) and rice (AY720933.1; 88%) proved that it was the exact sequence of the *EcCAS* gene.

**Figure 7:**
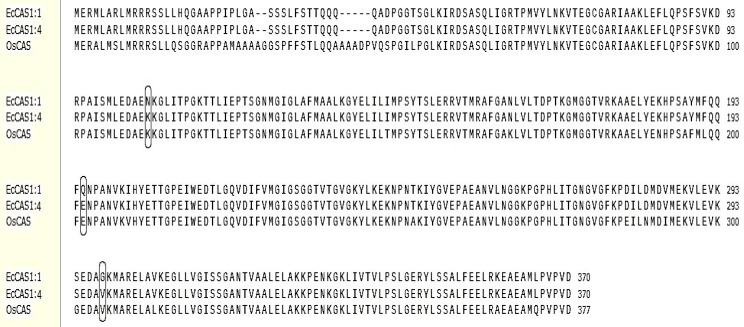
Open reading frame (ORF)-generated amino acid sequence of the *EcCAS* gene from quinclorac-resistant (*EcCAS*1:4) and susceptible (*EcCAS*1:1) *Echinochloa crus-galli* var. *mitis*. Three nucleotide mutations (Asn-105-Lys, Gln-195-Glu, and Gly-298-Val) among resistant and susceptible biotypes are indicated by a boxed codon. These mutations are similar to the rice *OsCAS* gene.

Nucleotide sequences were translated into amino acid sequences. The *EcCAS* gene from *E. crus-galli* var. *mitis* exhibited three and four copies in susceptible and resistant biotypes, respectively. *EcCAS*1;1, *EcCAS*1;2, and *EcCAS*1;3 were found in the FJ01-S biotype while *EcCAS*1;4, *EcCAS*1;5, *EcCAS*1;6, and *EcCAS*1;7 were found in all other biotypes. *EcCAS*1;1 and *EcCAS*1;4 were the most abundant copies in their respective biotypes whereas *EcCAS*1;2, *EcCAS*1;3, *EcCAS*1;5, *EcCAS*1;6, and *EcCAS*1;7 were detected in only 3–4 clones out of 24 clones sequenced in each biotype. EcCAS1;1 differed from *EcCAS*1;2 and *EcCAS*1;3 by one amino acid (Ile-Thr and Glu-Gln) at positions 269 and 138, respectively. *EcCAS*1;4 differed from *EcCAS*1;5, *EcCAS*1;6, and *EcCAS*1;7 at positions 29 (Gly-Asp), 117 (Glu-Gln), and 222 (Phe-Leu), respectively. Three mutations in nucleotide sequences were found in all copies of *EcCAS*1;4, *EcCAS*1;5, *EcCAS*1;6, and *EcCAS*1;7 as compared to three copies of FJ01-S. The mutations of AAC to AAA, CAG to GAG, and GGT to GTT resulted in amino acid substitutions of Asn to Lys, Gln to Glu, and Gly to Val at 105, 195, and 298 positions, respectively (Figure 7).

### 2.7. Effect of Mutation on β-CAS Structure and its Docking with PLP

**Figure 8:**
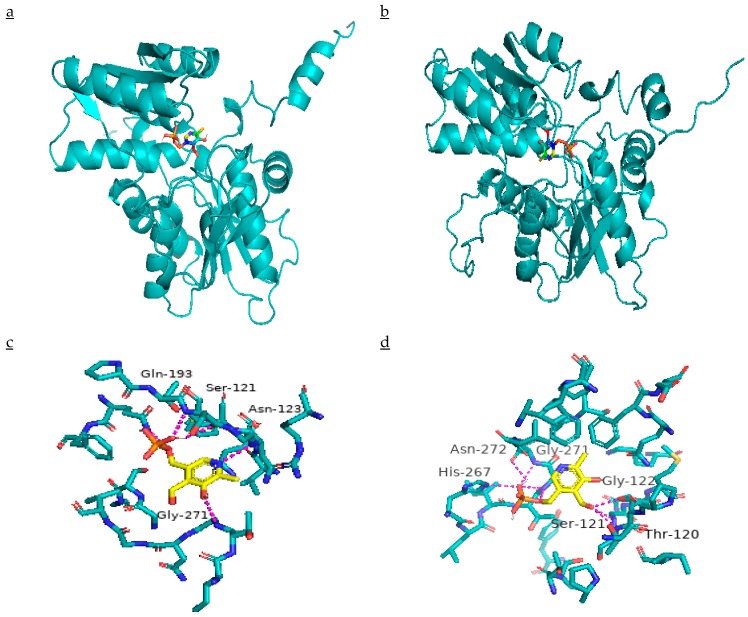
Ribbon diagram presenting the *β*-CAS structure of (**a**) quinclorac-susceptible, and (**b**) resistant *Echinochloa crus-galli* var. *mitis*. Molecular docking of PLP in active sites of (**c**) quinclorac-susceptible, and (**d**) resistant *β*-CAS.

Three amino acid mutations exhibited the change in ΔΔG (Gibbs free energy) of resistant *β*-CAS as compared to susceptible ones. Site-Directed Mutator (SDM) predicted ΔΔG values resulted due to Asn-105-Lys, Gln-195-Glu, and Gly-298-Val substitutions being 0.7, 0.01, and 0.72 kcal mol^−1^, respectively. The model parameters are given in Supplementary Table S1. According to SDM, ΔΔG > 0 kcal mol^−1^ indicates increased stability of structure due to mutation. Three-dimensional structures of *β*-CAS are shown in Figure 8a,b. The binding of PLP shows the active sites in the structure (Figure 8c,d). Three amino acid mutations affected the binding of PLP with *β*-CAS as PLP was bound with Ser-121, Asn-123, Gln-193, and Gly-271 amino acids in susceptible *β*-CAS, while in the resistant one, it was bound to Thr-120, Ser-121, Gly-122, His-267, Gly-271, and Asn-272. Molecular docking results revealed that binding free energy was decreased (−0.61 to −0.72 kcal mol^−1^) as the result of mutations that made resistant *β*-CAS to combine with its cofactor PLP more convenient.

### 2.8. Effect on the Expression Pattern of ACS and ACO Genes

**Figure 9:**
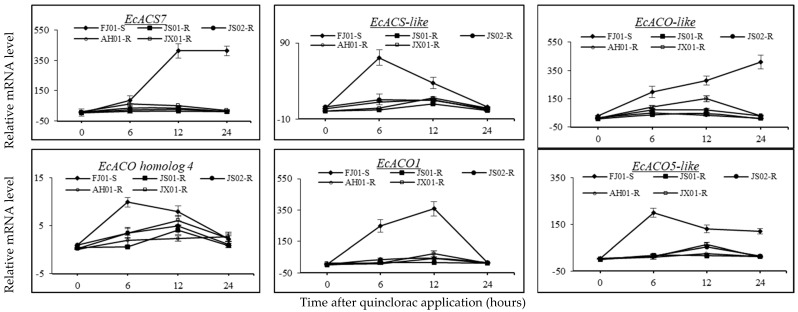
The ACS (*EcACS7*, *EcACS*-like) and ACO (*EcACO*-like, *EcACO homolog 4*, *EcACO*, *EcACO5*-like) genes relative expression levels in five biotypes of *Echinochloa crus-galli* var. *mitis* observed at 0, 6, 12, and 24 h after quinclorac application. *X*-and *Y*-axis represents the time after quinclorac application and relative transcription level to susceptible biotype (0 h, prior to quinclorac application), respectively. Data presented are the mean of three biological and four technical replicates. Vertical bars above mean represent the standard error. An underlined gene name represents upregulation of more than 10-fold.

The relative expression levels of *ACS* and *ACO* genes were studied at 0, 6, 12, and 24 HAT showed that the JS01-R did not express the significant increase in relative mRNA levels of all genes under study (Figure 9). Nonetheless, *EcACS7*, *EcACS*-like, *EcACO1*, *EcACO*-like, and *EcACO5*-like relative expression levels showed a remarkable upregulation as compared to 0 HAT in the FJ01-S biotype. The relative expression levels of these five genes were upregulated by 15-, 16-, 47-, 6-, and 37-fold at 6 HAT in FJ01-S. Furthermore, the upregulation of these genes (*EcACS7*, *EcACS*-like, *EcACO1*, *EcACO*-like, and *EcACO5*-like) in FJ01-S was 77-, 8-, 68-, 12- and 24-fold at 12 HAT, respectively, compared to 0 HAT. Expression levels of *EcACS*-like and *EcACO5*-like peaked at 6 HAT, while in the case of *ECACO1*, at 12 HAT. A decline in expression levels of the aforementioned genes was observed in later hours. *EcACS7* and *EcACO*-like did not show any decrease in relative expression levels until 24 HAT, as these were upregulated by 77- and 13-fold, respectively, compared to 0 HAT in FJ01-S (Figure 9).

Relative expression levels of *EcACS7*, *EcACS*-like, *EcACO1*, *EcACO*-like, and *EcACO5*-like were increased slightly in JS02-R, AH01-R, and JX01-R. Nevertheless, they were significantly lower than the increase in FJ01-S. A slight induction of expression levels was observed in *EcACO homolog 4* in FJ01-S and JX01-R biotypes. Relative transcript levels peaked at 6 HAT in FJ01-S and relatively maintained till 12 HAT, while the relative expression level in JX01-R peaked at 12 HAT and decreased after.

### 2.9. Effect on Transcript Level of EcCAS Gene

The relative transcript level of *EcCAS* observed from *E. crus-galli* var. *mitis* depicted that untreated resistant biotypes have higher (*p* < 0.05) relative mRNA levels as compared to control as the 1.4–2.6-fold increase was observed (Figure 6b). The quinclorac application resulted in a decline of *EcCAS* transcript levels in the susceptible biotype (FJ01-S) as, at 24 HAT, it decreased to half of that of the untreated plants. *EcCAS* transcript level was maintained by resistant biotypes till 24 HAT and did not increase significantly as compared to the untreated plants. At 12 HAT, the transcript level in the resistant biotypes depicted a 1.73–4.32-fold increase as compared to the susceptible biotype (Figure 6b).

## 3. Discussion

A long history of using quinclorac in rice fields can be held responsible for the quinclorac resistance outbreak in *Echinochloa* species. Quinclorac resistance was confirmed in *E. crus-galli* var. *mitis*. Misuse of quinclorac led to the evolution of resistance in these biotypes against this herbicide. As it is proposed earlier, the misuse of herbicide can lead to resistant weed species [25,26,28,40,45].

Quinclorac (auxin-type herbicide) is held responsible for ACS de novo synthesis initiation, ultimately leading to ethylene level increment and an upsurge in cyanide content in susceptible plants [18,30,46,47]. Ethylene (major plant hormone) is responsible for plant death and senescence [48]. Enzymes responsible for energy metabolism and respiration (cytochrome c oxidase in mitochondria) were severely affected by increased cyanides in quinclorac-susceptible biotypes [33,49]. Ethylene biosynthesis inhibition has been reported as a possible mechanism for quinclorac resistance in barnyard grass [18,19,38,47,50]. In the present study, a distinction in ethylene levels between resistant and susceptible strengthen the fact that quinclorac resistance is linked with ethylene biosynthesis inhibition, as it was formerly documented for other species in the literature [18,19,30,47]. In this study, quinclorac caused growth inhibition by increasing ethylene concentration in FJ01-S, which confirms the positive correlation between growth inhibition and ethylene production (Figure 4a). Therefore, the role of higher ethylene concentration in quinclorac phytotoxicity is validated [18,22,30,47,51].

ACS and ACO gene families were found controlling ethylene biosynthesis [34,52]. Auxin is responsible for the induction of twelve ACS genes found in *Arabidopsis* [53]. Previously, it was found that the increase in ethylene biosynthesis was related to the upregulation of ACS and ACO genes in quinclorac sensitive *E. crus-galli* var. *zelayensis* [19]. In the present study, different relative expression levels of *EcACS7*, *EcACS*-like, *EcACO1*, *EcACO*-like, *and EcACO5*-like genes were observed in quinclorac-resistant and susceptible *E. crus-galli* var. *mitis*. Changes in expression levels and changes in herbicide target tertiary structure can cause resistance [12,53]. This study presented that expression levels of *EcACS7*, *EcACS*-like, *EcACO1*, *EcACO*-like, and *EcACO5*-like a showed >10-fold increment in FJ01-S (quinclorac-susceptible biotype), while relative expression induction was lower in resistant biotypes.

ACC (precursor of ethylene) is converted from SAM by the activity of ACS; then, it is converted into ethylene (by the ACO activity) [23]. Considering the role of quinclorac in ethylene biosynthesis induction, ACC content, ACS, and ACO activities were mandatory for the quinclorac resistance mechanism study. Alongside this, *β*-CAS activity was essential to study to explore the cyanide detoxifying ability of resistant biotypes. In the present study, quinclorac application induced a higher increase in ACC, ACS, and ACO activities in FJ01-S as compared to resistant biotypes. Consequently, low ethylene production was recorded in resistant biotypes than susceptible ones. Previous studies also documented that the increase in ACC, ACS, and ACO activities was higher in quinclorac-susceptible biotypes than resistant ones [18,30,44,46,47]. In the present study, quinclorac-resistant biotypes exhibited a higher *β*-CAS activity as compared to susceptible ones; hence, cyanides produced after quinclorac application were detoxified more efficiently by these biotypes. Low *β*-CAS activity in the susceptible biotype resulted in cyanide accumulation in plants. Moreover, accumulated cyanides can trigger the ACS activity, thus causing more production of ethylene and cyanide [44]. So, cyanide degradation ability by *β*-CAS activity in resistant biotypes helped them to escape from more ethylene production by triggering the ACS. Down-regulation of *EcCAS* transcript levels in the susceptible biotype and maintenance of relative mRNA levels in the resistant ones as a result of quinclorac application support the positive correlation of quinclorac resistance with *β*-CAS activity. It can be inferred that ethylene response pathway alteration, as well as cyanide degradation, were responsible for quinclorac resistance in *E. crus-galli* var. *mitis*, as found in previous studies regarding *E. crus-galli* var. *zelayensis* [18,37]. In the present study, quinclorac resistance might be caused by decreased ACS and ACO activities in resistant biotypes, as quinclorac-susceptible biotype exhibited an increase in ACS (2–3 times) and ACO (2–4 times) activities than resistant ones. These results strengthen the role of ethylene biosynthesis inhibition conferring quinclorac resistance.

The molecular study of the *EcCAS* gene exhibited that there were three mutation points causing Asn-105-Lys, Gln-195-Glu, and Gly-298-Val substitutions in resistant biotypes as compared to susceptible ones. Differential gene expression and mutations can cause non-target enzyme activity [15,54]. Altered gene expression as a result of mutations can be held responsible for herbicide resistance [11,12]. The *EcCAS* amino acid sequence analyzed on the website http://www.ebi.ac.uk/interpro/, revealed that mutations at 105, 195, and 298 positions are part of a domain (Domain ID IPR001926) and it consisted of 286 amino acid. Its molecular function is L-3-cyanoalanine synthase activity and cysteine synthase activity, and it involves biological processes like cyanide metabolic process and cysteine biosynthetic process from serine. In resistant biotypes, Asn-105-Lys, Gln-195-Glu, and Gly-298-Val substitution lying motifs *α*1, *β*6B, and *β*9A [35], respectively, were found similar to rice (quinclorac tolerant) [36]. The amino acid sequence comparison with other species *CAS* gene sequences revealed that these mutations were in accordance with wheat and most of the other species at the same nucleotide positions (Supplementary Figure S1). PLP working as cofactor binds *β*-CAS with cysteine; this repositioned the amino acid to be attacked by CN^−^ and led to the production of *β* cyanoalanine [55]. Molecular docking of PLP with *β*-CAS provided an insight of bonding alteration among quinclorac-resistant and susceptible *β*-CAS enzymes. By making polar bonds with six amino acid subunits in the active site, resistant *β*-CAS depicted lower binding free energy, hence making bonding easier and more firm than susceptible ones. All biological processes in cells are regulated by protein–protein interactions. Protein affinity and structure stability can severely be affected by mutations in protein, resulting in a malfunction in the network of protein interaction [56]. *β*-CAS enzyme structure stability predicted by SDM depicted that these three mutations are not contributing to structure destabilization; hence, these play a role in stabilizing the structure of the enzyme. On the other hand, single nucleotide polymorphism (SNP) in the *β*-CAS gene of quinclorac-resistant *E. phyllopogan* as compared to the susceptible biotype was found to be not correlated with quinclorac resistance [38]. The reported mutation occurred at intron and quinclorac sensitive biotypes lacking that mutation caused alternative gene splicing. Nevertheless, the mentioned SNP did not cause any change in the translated amino acid sequence, so increased *β*-CAS activity due to the prevention of alternative splicing may not be correlated with quinclorac resistance. However, in the present study, three-point mutations were found in the *EcCAS* gene of quinclorac-resistant *E. crus-galli* var. *mitis*, which resulted in amino acid substitutions. It is a fact that HCN produced during ethylene biosynthesis is degraded by *β*-CAS enzyme activity [35]. So, the mutation in the respective gene helped to maintain enzymatic activity even after the quinclorac application. Moreover, *β*-CAS activity can still be correlated to quinclorac resistance. Mutations in Arabidopsis Starch Synthase III were found to increase enzyme expression level and its activity [57]. Numerous studies documented that mutations in gene resulted in increased enzyme activity [39,58]. In the present study, molecular simulation results revealed that these mutations are favoring the bonding between the enzyme and its cofactor by stabilizing the structure, decreasing the binding free energy, and increasing its binding site residues. Mutations can increase the affinity of the enzyme to its substrate [59]. Moreover, mutations can lead to structural stability [60]. This can be inferred that these mutations in the *EcCAS* gene proved to be favorable in terms of increased *β*-CAS enzyme activity.

So, keeping in the context of these results, less inducible expression of *EcACS7*, *EcACS*-like, *EcACO1*, *EcACO*-like, and *EcACO5*-like genes resulted in a decrease of ACS and ACO activities. On the other hand, the higher *β*-CAS activity, the lower binding free energy of *β*-CAS enzyme, and mutations in the *EcCAS* gene resulting in its differential transcript levels among resistant and susceptible biotypes, ultimately leading to reduced ethylene biosynthesis coupled with improved cyanide degradation, hence causing quinclorac resistance.

## 4. Materials and Methods

### 4.1. Plant Material

Seeds of *E. crus-galli* var. *mitis* were collected from different rice fields of Jiangsu (Funing 33°76′ N/119°93′ E; Yixing 31°36′ N/119°63′ E), Anhui (Dongzhi County 30°56′ N/117°59′ E), Jiangxi (Yifeng 28°38′ N/115° 81′ E), and Fujian (Pucheng, 27°80′ N/118°44′ E) provinces in China during fall 2016 (Supplementary Figure S2). These two sites from Jiangsu province and the latter two ones were subjected to quinclorac application for more than 15 years and 10 years, respectively, except for the Pucheng site. Seeds were shade dried and stored at room temperature. These seeds were examined for a different level of quinclorac in a Petri plate assay. A susceptible biotype (S) from Fujian province (Pucheng, 27°80′ N/118°44′ E) was used for comparative analysis. Seeds of all biotypes were planted in the field (30 and 50 plants for R and S biotypes, respectively). The seedlings at 3–4 leaf stage were subjected to a quinclorac field dose (300 g a.i. ha^−1^), and half of the S seedlings were not sprayed. The inflorescence of each plant was covered (35 days after emergence) with a butter paper bag to avoid the cross-pollination between different biotypes. F_1_ generations of four surviving biotypes of the quinclorac field dose were further used in the study and were designated as resistant biotypes (R). The F_1_ generation of S seedlings that were not sprayed with quinclorac was further used in the study. All five biotypes were named as per their province names: JS01-R, JS02-R, AH01-R, JX01-R, and FJ01-S (R and S representing the resistant and susceptible biotypes).

### 4.2. Chemical

Quinclorac used in the present study was purchased from Jiangsu Futian Agricultural Chemicals (Nanjing, China). Poly (vinylpolypyrrolidone), polyvinylpyrrolidone, Dithiothreitol (DDT), Tricine, and SAM were bought from Solarbio Science and Technology Co. Ltd. (Beijing, China). Sodium bicarbonate, ACC, and MOPS were purchased from MDBio Inc (Taipei, Taiwan). 5-Sulfosalicylic acid dehydrate, and sodium hypochlorite were obtained from BBI Life Science and Sangon Biotech (Shanghai, China).

### 4.3. Quinclorac Resistance

#### 4.3.1. Petri Plate/Seed Bioassay

Seeds of R and S biotypes were incubated on wet filter papers (Whatman No. 1; Sigma-Aldrich, St. Louis, MO, USA) containing five varying concentrations of quinclorac (1200, 600, 300, 150, 75 mg L^−1^), along with quinclorac free control (distilled water). Plastic Petri plates (9 cm diameter) were purchased from Shanghai Joylab Medical Instruments Co. Ltd. (Shanghai, China) and used in the study, each containing 15 seeds of either biotype. Each Petri plate was irrigated with either 5 mL herbicide solution or distilled water as per treatment and rehydrated when needed (approximately 3 days interval) with distilled water. The experiment was conducted in a growth chamber set at 60% relative humidity; day/night period was 14/10 h having 8000 lux light intensity, and 26/18 °C temperature. The shoot length of five random seedlings per Petri plate was measured after 9 days and averaged after. The experiment was conducted twice with three replications per treatment.

#### 4.3.2. Resistance Confirmation in Pot Assay

For whole-plant bioassay, seeds of each biotype were grown in a plastic pot (12 cm diameter) containing a 2:1 mixture of sand and organic soil (NPK ≥2%, organic matter ≥35%, and pH = 5.5–6.5) purchased from Zhenjiang Peilei Organic Fertilizer Co. Ltd. (Zhenjiang, China). Pots were placed in watered containers to provide sub-irrigated conditions for plant growth. Growth conditions were the same as described in Section 4.3.1, and 5 seedlings were maintained per pot. At 3–4 leaf stage, quinclorac was sprayed with a laboratory sprayer equipped with a flat fan nozzle (3WPSH-500D, Nanjing, China), with a delivering capacity of 280 L ha^−1^ at 230 kPa [18]. Quinclorac dose rates were 0, 75, 150, 300, 600, and 1200 g a.i. ha^−1^ for R biotypes and 0, 37.5, 75, 150, 300, and 600 g a.i. ha^−1^ for S biotype. Above ground plant parts were clipped after three weeks of herbicide spray, and fresh weight was measured. The experiment was conducted twice using three replications for each treatment.

### 4.4. Ethylene Production by R and S Biotypes

To find the quinclorac resistance in R biotypes associated with ethylene biosynthesis inhibition, an experiment was conducted having four replicates. Quinclorac with four varying concentrations (0, 150, 300, 600 g a.i. ha^−1^) was sprayed on R and S biotypes at 3–4 leaf stage. Plant shoots from each treatment were clipped at varying time intervals (0, 12, 24, 48, 96 h after spray) and incubated in a 15 mL tarred screw-neck vial for five hours at 25 °C. The quantitative determination of ethylene was carried out by Abdallah’s method with a few modifications [18]. After incubation, a syringe was used to withdraw 1 mL gas and injected into GC system 7890A (Agilent Technologies, Santa Clara, CA, USA) equipped with a column and flame ionization detector to measure the ethylene quantity. The temperature was set at 70, 200, and 250 °C for injector, detector, and column, respectively. The flow rates were 30, 300, and 30 mL min^−1^ for N_2_, air, and H_2_, respectively. The ethylene contents were measured in nmol g^−1^ fresh weight h^−1^ of incubation. Another set of the same experiment was run alongside and shoot fresh weight was measured 7 days after spray and expressed as percent of mean untreated control.

### 4.5. Effect on Endogenous ACC Levels

An experiment was carried out to find out whether quinclorac application attributed to the difference in ACC levels of R and S biotypes. The dose of quinclorac and sampling times were the same as for ethylene quantitative determination. ACC (nmol g^−1^ plant fresh weight) contents were measured as described earlier [45,61] with some modifications [62]. After powdering in liquid N_2_, 0.2 g shoot tissue was extracted in 0.4 mL 5% sulfosalicylic acid [62]. ACC in samples was chemically converted into ethylene, HgCl_2_ was used to stop the conversion of ACC into ethylene before its measurement by GC system.

### 4.6. Effect on ACS Activity

ACC (precursor for ethylene biosynthesis) is transported to shoot from roots. So, the activity of ACS is mainly confined in roots [46]. Hence, ACS activity (pmol g^−1^ root fresh weight h^−1^ of incubation) was measured from root tissues at 24 h after treating the plants with quinclorac (0, 150, 300, 600 g a.i. ha^−1^) by following the Grossmann methods [44,46]. The crushed frozen sample of 0.5 g was centrifuged with extraction solution (100 mM potassium phosphate buffer (pH 8.5), 10 μM leupeptin, 6 μM PLP, and 5 mM DTT) at 15,000× *g* at 4 °C for 15 min. The supernatants were eluted from Sephadex G- 25 desalting columns after equilibration with 5 mM potassium phosphate buffer containing 6 μM PLP and 1 mM DTT. Enzyme preparation (0.3 mL) was incubated for 2 h at 37 °C with ACS assay mixture (20 μM PLP and 100 μM SAM in 80 mM potassium phosphate buffer (pH 8.5)). Lastly, the reaction was stopped by adding 20 μmol HgCl_2_. During the incubation time, ACS activity was investigated by monitoring ACC levels. When the reaction was quenched at time zero, the background level of ACC could be determined by its conversion into ethylene.

### 4.7. Effect on ACO Activity

ACC is converted to ethylene by the activity of ACO. Plants were treated with quinclorac (0, 150, 300, and 600 g a.i. ha^−1^) to find out whether there is a difference between R and S biotypes regrading ACO activity (pmol g^−1^ root fresh weight h^−1^ of incubation). Shoot samples were collected 24 h after spray. ACO activity was measured following the method of Bulens [62]. Polyvinylpolypyrrolidone (50 mg), 0.5 g crushed frozen sample, and 1 mL MOPS extraction buffer (400 mM, pH 7.2), MOPS, glycerol (10% *v*/*v*), and ascorbic acid sodium salt (30 mM) were vortexed until a homogenous mixture was obtained. Samples were incubated in thermomixer for 10 min at 4 °C, followed by centrifugation at 22,000 for 30 min at the same temperature. Supernatants were allowed to react with MOPS reaction buffer (50 mM, pH 7.2), MOPS, glycerol (10% *v*/*v*), ascorbic acid sodium salt (5 mM), sodium bicarbonate (20 mM), iron sulfate (0.02 mM), ACC (1 mM), and DTT (1 mM) in a glass vial having a rubber stopper on the water bath at 30 °C for 1 h. After incubation, headspace gas was withdrawn by syringe, and ethylene was measured by the GC system following the method described earlier. ACO activity of samples was calculated by subtracting the quantity of non-enzymatic ethylene production from reaction buffer.

### 4.8. Cyanide Detoxification by β-CAS

The *β*-CAS activity was determined to find the difference in cyanide detoxification by R and S biotypes. Germinated seeds of five biotypes were cultured in Kasugai nutrient solution (hydroponically) till 3–4 leaf stage. The growth conditions of the incubator were the same as described in Section 4.3.1. At this stage, quinclorac (50 μmol L^−1^ (μM)) was added to the nutrient solution. Plant samples were collected 0, 12, and 24 h after adding quinclorac. The *β*-CAS activity was determined by the previously described procedure [63]. Then, 0.1 M Tris-HCl buffer (pH 8.5) was used to extract the powdered shoots. The supernatant was used in enzyme analysis after centrifuging it for 10 min at 4 °C and 10,000× *g*. Enzyme extract (0.2 mL) was incubated at 35 °C for 30 min with the same extraction buffer containing 3 mM L-cysteine and 25 mM NaCN in a total volume of 1 mL. Using standard (Na_2_S), absorbance was measured at 650 nm after adding 100 µL of each 30 mM FeCl_3_ in 1.2 N HCl and 20 mM N, N-dimethyl-p-phenylenediamine in 7.2 N HCl.

### 4.9. EcCAS Gene Sequence

Partial sequence of *EcCAS* in *E. crus-galli* var. *mitis* was determined by using primers (F-CCGTCCTTCAGCGTCAAA, R- ATGCCAACCAAAAGACCCT, TM = 55 °C), designed based on *EcCAS* cDNA sequence from *E. crus-galli* var. *zaleyensis* (KY922855.1). Plant Easy Spin RNA Miniprep Kit (Biomiga, San Diego, CA, USA) was used for the total RNA extraction, while cDNAs were synthesized by PrimeScript™ RT Reagent Kit with gDNA Eraser (TaKaRa, Otsu, Japan). Partial amplification was carried out in total volume of 50 µL (TaKaRa ExTaq^TM^ 0.4 µL, 10× Taq Buffer 5 µL, MgCl_2_, and dNTP 4 µL each, cDNA, forward and reverse primers 2 µL each, ddH_2_O 30.6 µL) using TaKaRa PCR Thermal Cycler (TaKaRa, Otsu, Japan) with PCR profile consisting of denaturation at 94 °C for 4 min, then 28 cycles at 94 °C, 55 °C, and 72 °C for 30, 30, and 60 s, respectively, followed by 10 min at 72 °C. The PCR product was gel-purified by Gel/PCR Extraction Kit (Biomiga, San Diego, CA, USA). The purified cDNA was cloned in E. coli DH5*α* Competent Cells (Clontech, Mountain View, CA, USA) by pMD^TM^ 19-T Vector Cloning Kit (Clontech, Mountain View, CA, USA) and sent to Sangon Biotech (Shanghai, China) for sequencing.

Rapid Amplification of cDNA Ends (RACE) was carried out using SMARTer^®^ RACE 5′/3′ Kit (Clontech, Mountain View, CA, USA) using the primers (5′-RACE-CCCTTKTTTTCAGCATCTTCCAACATTGAA, 3′-RACE-AAAGTGAGGATGCTGKTAAGATGGCTCGAGA, TM =< 70 °C) and following the instructions provided by the company. PCR product was gel purified and sent to the company for sequencing. Sequences were aligned by using BioEdit Sequence Alignment Editor [64]. To find the ORF of the *EcCAS* gene. the ORF finder function was used on the NCBI website (https://www.ncbi.nlm.nih.gov/orffinder/). Then, it was amplified by primers (F- CACACACAAAAACAGCGAGT, R- ACACAGTTGCCAGGAAGTTT, TM = 54 °C) and sequenced (4 individual plants and 6 clones from each replicate) from the company following the same procedure as described above.

### 4.10. Computational Study of β-CAS Binding with PLP and Its Structure Stability

Homology modeling of quinclorac-resistant and susceptible *β*-CAS structure was carried out using 3VBE as a template protein. Protein Homology/Analogy Recognition Engine (Phyre^2^) [65] was used to construct the 3D structure. The PLP structure was downloaded from PubChem (CID: 1051). AutoDock Tools v. 1.5.6 (Molecular Graphics Laboratory, The Scripps Research Institute, San Diego, CA, USA) was used to prepare both structures for docking. Docking of *β*-CAS and PLP was carried out using AutoDock Vina v. 1.1.2 (Molecular Graphics Laboratory, The Scripps Research Institute, San Diego, CA, USA). Docking grid box size (Å) was X:44.36, Y:70.57, and Z:54.19 for the quinclorac-susceptible *β*-CAS enzyme, and X:63.35, Y:51.03, and Z:44.69 for the resistant ones. Docking results were viewed and extracted by PyMOL v. 2.3.3 (Schrödinger, Inc New York, NY, USA). SDM [66] was employed to compute the Gibbs free energy {ΔΔG_susceptible→resistant_(kcal mol^−1^} of the *β*-CAS enzyme structure.

### 4.11. Real-Time PCR Based Expression Analysis of ACS, ACO, and CAS Genes

Seeds of five biotypes were grown as described in Section 4.8. Plant samples were collected 0, 6, 12, and 24 h after adding quinclorac and stored at −70 °C until further analysis. Six genes (four ACO and two ACS) were selected based on the previous study on *E. crus-galli* var. *zaleyensis* [19] as well as the *EcCAS* gene (Genbank accession no. MN815009 and MN815010) sequenced in the present study. cDNA sequence of *EcACS7* (KY963550), *EcACS*-like (KY963549), *EcACO*-like (KY963552), *EcACO homolog 4* (KY963556), *EcACO1* (KY963554), and *EcACO5*-like (KY963548) were obtained from the NCBI website (www.ncbi.nlm.nih.gov/nuccore/) and further used for primer designing (Table 4). The reference gene used in this study was *EcActin* (HQ395760; Table 4). cDNA was synthesized as described in Section 4.9. Gene expression analysis of five biotypes of *E. crus-galli* var. *mitis* was performed by using SYBR^®^ Premix Ex Taq^TM^ (Tli RNAaseH Plus; TaKaRa, Otsu, Japan) on real-time PCR (Eppendorf Mastercycler^®^ Realplex^2^, Eppendorf North America Inc, Hauppauge, NY, USA). The reaction was conducted in a total volume of 10 µL, containing 5 µL SYBR^®^ Premix Ex Taq^TM^, 0.4 µL primers, 0.8 µL cDNA (adjusted to the same concentration), and 3.8 µL ddH_2_O. PCR profile was as follows: 95 °C for 30 s, 40 cycles at 95 °C for 5 s, and 60 °C or 30 s. Relative transcript levels were calculated by the ΔΔCt method [67] with three biological and four technical replicates. Up- or down-regulation of gene expressions triggered by quinclorac application was determined by the *t*-test (*p* < 0.05) and fold change (threefold).

### 4.12. Statistical Analysis

Dose–response assays (Petri plate and pot assay) were conducted twice in triplicate. The experiments related to ethylene concentration, ACC contents, ACS, ACO, and β-CAS activities were conducted twice in quadruplicate. A completely randomized design was used for all experiments. Percent of shoot length/fresh weight reduction in relation to the untreated control was calculated and further used for calculations. EC_50_ (Petri plate assay) and GR_50_ (pot assay) values (effective concentration to inhibit growth by 50%) were calculated in Origin Pro 8 SR0 (v. 8.0724 (B724), Origin Labs Corporations. MA 01060. USA. http/:www.originlab.com) by using the dose–response function (growth/sigmoidal).

y=A1+A2−A11+10^(logx0−x)p

where A1 and A2 are bottom and top asymptote, respectively, X is quinclorac dose, Xo is quinclorac dose required to cause 50% growth inhibition, and *p* is the slope.

Resistance level was calculated by dividing the GR_50_ of each R population with GR_50_ of S population. EC_50_ ratio is the ratio between EC_50_ of each R population with S population. Primer Premier 5 (PREMIER Biosoft International, San Francisco, CA, USA) was used to design primers. Microsoft Excel 2019 was used to draw the graphs. SPSS version 24 (SPSS, Chicago, IL, USA) was used for Pearson correlation analysis and to construct ANOVA.

## 5. Conclusions

Vivid induction of expressions of *EcACS7*, *EcACS*-like, *EcACO1*, *EcACO*-like, and *EcACO5*-like genes in quinclorac-susceptible biotype (FJ01-S) of *E. crus-galli* var. *mitis* after herbicide treatment gave rise to ACC synthase and ACC oxidase enzyme activities, which resulted in increased ethylene biosynthesis. On the other hand, the quinclorac-resistant biotype (JS01-R) did not show any remarkable increase in the relative expression of these genes. Hence, low activities of ACC synthase and ACC oxidase enzymes were observed, which led to low ethylene biosynthesis and possibly caused quinclorac resistance. FJ01-S depicted a decline in the transcript level of the *EcCAS* gene post-treatment with quinclorac; hence, cyanides produced during ethylene biosynthesis were accumulated due to lower *β*-CAS activity. Mutations in the *EcCAS* gene of resistant biotypes resulted in enzyme structure alteration, thus decreased binding free energy and helped them to maintain gene transcript levels as well as upsurge the *β*-CAS activity; hence, cyanide was degraded efficiently. The other biotypes were found less resistant as compared to JS01-R. This can be justified by a slight induction of *EcACS7, EcACS*-like, *EcACO1*, *EcACO*-like, and *EcACO5*-like gene expression. This caused a little increase in ACC synthase and ACC oxidase enzyme activities as well as ethylene levels. However, this overall increase was far lower than in the FJ01-S biotype, hence causing a moderate quinclorac resistance in these biotypes. However, further investigations are required, whether quinclorac induces a lesser amount of ethylene first or directly inducing the *EcACS7*, *EcACS*-like, *EcACO1*, *EcACO*-like, and *EcACO5*-like genes upregulation by acting as a signaling substance.

## Figures and Tables

**Table 1:** EC_50_, GR_50_, and RI of quinclorac-resistant and sensitive biotypes of *Echinochloa crus-galli* var. *mitis*.

Populations	Dose–Response Analysis					
	EC_50_ (mg L^−1^)	*r* ^2^	RI (EC_50_ Ratio)	GR_50_ (g a.i. ha^−1^)	*r* ^2^	RI (GR_50_ Ratio)
**FJ01-S**	118.69 (7.82)	0.98	1	26.83 (1.02)	0.999	1
**JS01-R**	4116.36 (76.65)	0.99	35	733.73 (26.96)	0.999	27
**JS02-R**	2604.65 (35.61)	0.99	22	447.1 (14.73)	0.999	17
**AH01-R**	2953.82 (37.78)	0.96	25	484.22 (19.42)	0.98	18
**JX01-R**	1605.25 (12.56)	0.98	14	313.82 (11.08)	0.99	12

EC_50_ = effective dose responsible for 50% inhibitory response, GR_50_ = effective dose responsible for 50% inhibition in growth, RI (Resistance Index) = ratio of EC_50_ R over EC_50_ S or GR_50_ R over GR_50_ S, *r*^2^ = coefficient, *p* < 0.05. Standard errors are in parenthesis.

**Table 2:** ACS activity (pmol g^−1^ fresh weight h^−1^) exhibited by different biotypes of *Echinochloa crus-galli* var. *mitis* after 24 h of quinclorac treatment (0, 150, 300, 600 g a.i. ha^−1^).

Dose (g a.i. ha^−1^)	Populations				
	FJ01-S	JS01-R	JS02-R	AH01-R	JX01-R
**0**	54.51 ± 2.55 ^cA^	40.65 ± 2.99 ^aB^	45.33 ± 2.10 ^cAB^	42.74 ± 1.06 ^bB^	44.73 ± 3.10 ^cAB^
**150**	69.81 ± 4.19 ^cA^	44.69 ± 3.14 ^aC^	51.71 ± 1.03 ^bcBC^	49.72 ± 2.86 ^abBC^	58.28 ± 2.66 ^bAB^
**300**	86.20 ± 3.01 ^bA^	46.10 ± 2.44 ^aD^	58.37 ± 3.34 ^abC^	52.83 ± 1.26 ^aCD^	69.54 ± 1.48 ^aB^
**600**	120.69 ± 4.64 ^aA^	49.13 ± 1.57 ^aD^	66.42 ± 2.13 ^aBC^	56.26 ± 2.25 ^aCD^	76.43 ± 1.70 ^aB^

Data are means ± standard errors of four replicates. ^a–c^ showing the significant difference between different doses in one population, ^A–D^ representing significant difference among different populations under the same dose. ACS = 1-aminocyclopropane−1-carboxylic acid synthase.

**Table 3:** ACO activity (pmol g^−1^ fresh weight h^−1^) exhibited by different biotypes of *Echinochloa crus-galli* var. *mitis* after 24 h of quinclorac treatment (0, 150, 300, 600 g a.i. ha^−1^).

Dose (g a.i. ha^−1^)	Populations				
	FJ01-S	JS01-R	JS02-R	AH01-R	JX01-R
**0**	303.93 ± 8.58 ^dA^	158.72 ± 16.33 ^cC^	222.29 ± 24.77 ^bBC^	192.76 ± 7.33 ^cBC^	240.43 ± 18.53 ^dAB^
**150**	538.21 ± 21.05 ^cA^	204.82 ± 8.95 ^bC^	303.31 ± 18.52 ^abB^	222.87 ± 5.51 ^bcC^	322.69 ± 4.18 ^cB^
**300**	749.73 ± 18.69 ^bA^	230.83 ± 5.45 ^bD^	340.32 ± 37.29 ^aBC^	267.16 ± 12.92 ^bCD^	412.75 ± 27.77 ^bB^
**600**	1055.19 ± 22.68 ^aA^	276.18 ± 5.09 ^aD^	403.30 ± 13.61 ^aC^	369.14 ± 15.60 ^aC^	603.23 ± 5.89 ^aB^

Data are means ± standard errors of four replicates. ^a–d^ showing the significant difference between different doses in one population, ^A–D^ representing significant difference among different populations under the same dose. ACO = 1-aminocyclopropane-1-carboxylic acid oxidase.

**Table 4:** Primers sequence used for the amplification of *ACS*, *ACO,* and *CAS* genes in *Echinochloa crus-galli* var. *mitis*.

Gene	Forward Primer (5′-3′)	Reserve Primer (5′-3′)	Annealing Temperature (°C)	Length of Product
*EcActin*	TACTCCTTCACCACAACCGC	TGATGACCTGTCCATCAGGC	60	154
*EcACS7*	TCGCGAGGATGAGCAGATTC	ACCCAAGGTAGTATTATTTACCCTC	60	124
*EcACS*-like	GATGCTGTCGGACCACGAG	GTCCATCCACGAGAAGAGCC	60	148
*EcACO homolog 4*	CATCTTCCCGCACACGGAC	GATGGAAACCCTTGGCTCGT	60	250
*EcACO1*	AGTCCCAGGTTTGGAGTTTCTG	ATTATGGCGTCAGCACCAGG	60	207
*EcACO*-like	CCGGAGTTCAAGGAGACCAT	TGACCTTGGTGCCGAAGAAG	60	163
*EcACO5*-like	GCACATGGCGGTGAACTACT	CACGCTCTTGTAGCGGTCAT	60	229
*EcCAS*	TGCCGTCATACACCAGTCTT	CCAATCCCCATCACARAAA	56	253

## Supplementary Materials

Supplementary materials can be found at https://www.mdpi.com/1422-0067/21/5/1573/s1.

## Abbreviations

ACC	1-aminocyclopropane-1-carboxylic acid
*ACS*	1-aminocyclopropane-1-carboxylic acid synthase
*ACO*	1-aminocyclopropane-1-carboxylic acid oxidase
*β*-CAS	*β*-cyanoalanine synthase
*EcCAS*	*Echinochloa crus-galli* var. *mitis* β-cyanoalanine synthase
Asn	Asparagine
Lys	Lysine
Gln	Glutamine
Glu	Glutamic acid
Gly	Glycine
Val	Valine
TSR	Target-site resistance
NTSR	Non-target-site resistance
Aux/IAA	*Auxin/Indole-3-Acetic Acid*
HCN	Hydrogen cyanide
PLP	Pyridoxal phosphate
ALS	Acetolactate synthase
EC_50_	Effective dose responsible for 50 % inhibitory response
GR_50_	Effective dose responsible for 50% inhibition in growth
RI	Resistance Index
HAT	Hours after treatment
ORF	Open Reading Frame
Ile	Isoleucine
Thr	Threonine
Asp	Aspartic acid
Phe	Phenylalanine
Leu	Leucine
SDM	Site-Directed Mutator
ΔΔG	Gibbs free energy
SAM	S-Adenosyl-L-methionine
CN^−^	cyanides
SNP	Single nucleotide polymorphism
MOPS	3-(N-morpholino)propanesulfonic acid
DDT	Dithiothreitol
h	hours
S	Susceptible
R	Resistant
