# Supplementary material for: Ethylene Biosynthesis Inhibition Combined with Cyanide Degradation Confer Resistance to Quinclorac in Echinochloa crus-galli var. mitis
Source: Int J Mol Sci. 2020 Feb 25;21(5):1573. doi: 10.3390/ijms21051573 (PMC7084851; doi:10.3390/ijms21051573)
Supplement: Supplementary file 1 [file ijms-21-01573-s001.zip › Supplementary Material/S. Figure 1.docx]

| 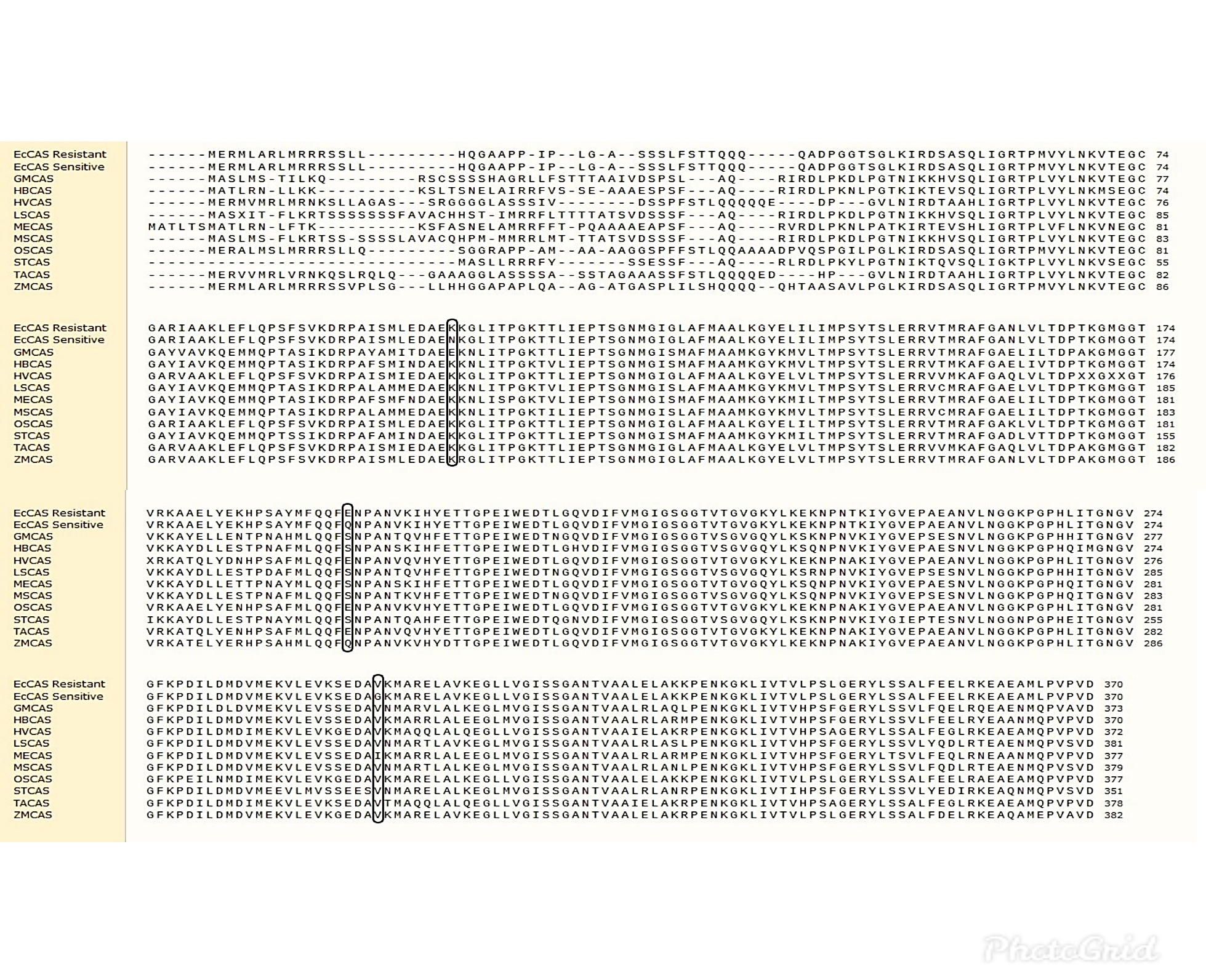 |
| --- |
| **Supplementary Figure S1.** Ec*CAS* amino acid sequence comparison with other plant species. Boxed amino acid sequences represent the point of mutation found in quinclorac resistant and sensitive *Echinchloa crus-galli* var. *mitis* (EcCAS resistant and EcCAS sensitive; Asn-105-Lys, Gln-195-Glu, and Gly-298-Val).  CAS = β-cyanoalanine synthase, GM = *Glycine max*, HB = *Hevea brasiliensis*, HV = *Hardium Vulgare*, LS = *Lathyrus sativus*, ME = *Manihot esculenta*, MS = *Medicage sativa*, OS = *Oryza sativa*, ST = *Solanum tuberosum*, TA= *Triticum aestivaum*, ZM = *Zea mays*. |
