# Supplementary material for: Ethylene Biosynthesis Inhibition Combined with Cyanide Degradation Confer Resistance to Quinclorac in Echinochloa crus-galli var. mitis
Source: Int J Mol Sci. 2020 Feb 25;21(5):1573. doi: 10.3390/ijms21051573 (PMC7084851; doi:10.3390/ijms21051573)
Supplement: Supplementary file 1 [file ijms-21-01573-s001.zip › Supplementary Material/S. Figure 2.docx]

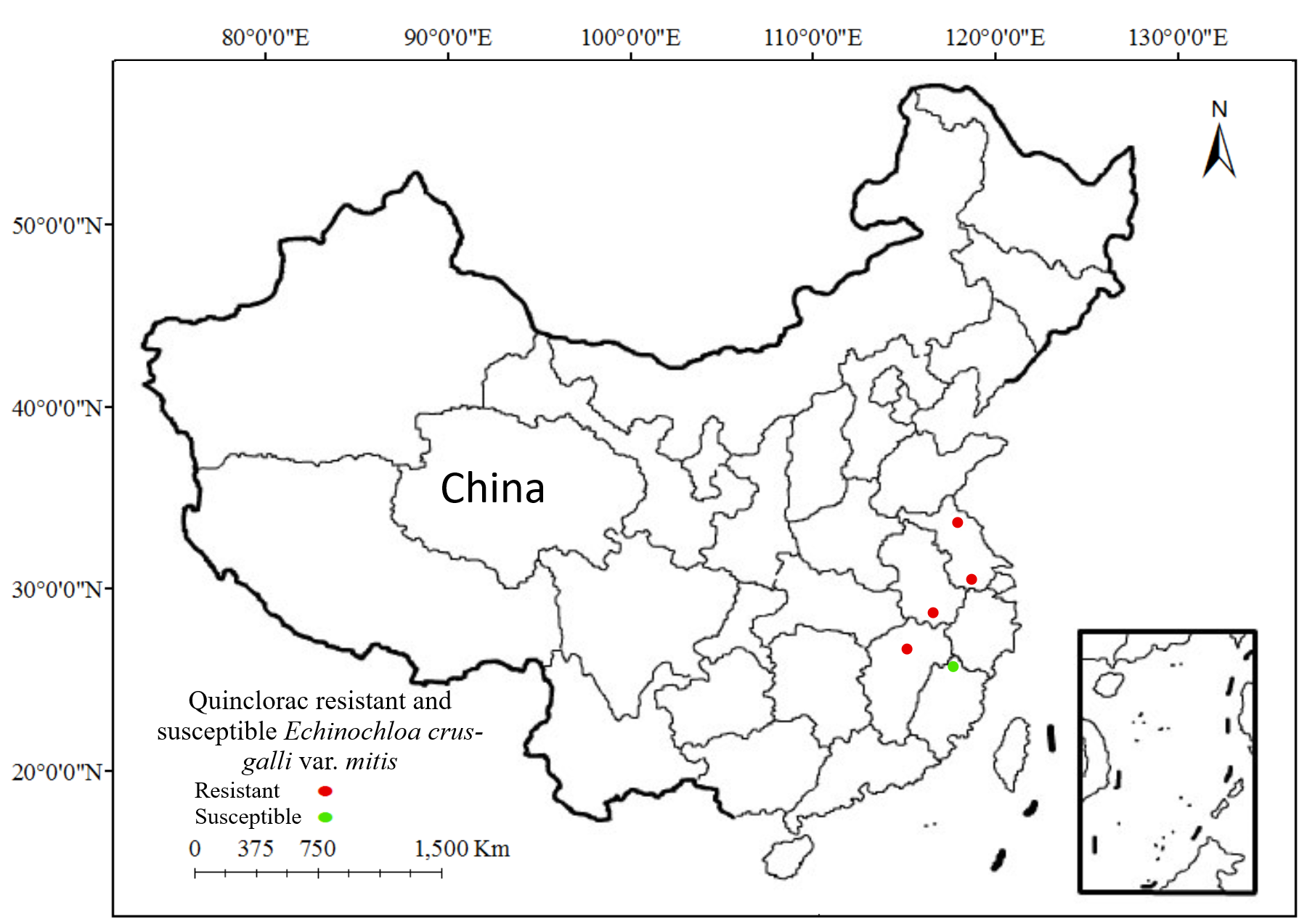


**Supplementary Figure S2.** The map of China indicating the collection sites of quinclorac resistant and susceptible *Echinochloa crus-galli* var. *mitis*. The susceptible biotype was collected from Fujian province, while resistant biotypes were found in Jiangsu, Anhui, and Jiangxi provinces.
