# Supplementary material for: Ethylene Biosynthesis Inhibition Combined with Cyanide Degradation Confer Resistance to Quinclorac in Echinochloa crus-galli var. mitis
Source: Int J Mol Sci. 2020 Feb 25;21(5):1573. doi: 10.3390/ijms21051573 (PMC7084851; doi:10.3390/ijms21051573)
Supplement: Supplementary file 1 [file ijms-21-01573-s001.zip › Supplementary Material/S. Table 1.docx]

**Supplementary Table S1:** Model parameters of SDM

| **Mutation** | **Wild Type** | | | **Mutant** | | | **Predicted ΔΔG (kcal mol^-1^)** | **Outcome** |
| --- | --- | --- | --- | --- | --- | --- | --- | --- |
|  | **RSA%** | **Depth Å** | **OSP** | **RSA%** | **Depth Å** | **OSP** |  |  |
| Asn-105-Lys | 95.8 | 3.2 | 0.17 | 84.5 | 3.3 | 0.14 | 0.70 | Increased stability |
| Gln-195-Glu | 54.5 | 3.5 | 0.26 | 61.7 | 3.4 | 0.24 | 0.01 | Increased stability |
| Gly-298-Val | 30.1 | 5.6 | 0.50 | 14 | 4.3 | 0.51 | 0.72 | Increased stability |

RSA = relative sidechain solvent accessibility, Depth of a residue is defined as the average distance of all-atom depths found in the residue from the nearest surface water molecule., OSP = residue-occluded packing density
